# Measuring beliefs about free will: a critical review of the Free Will Inventory

**DOI:** 10.3389/fpsyg.2026.1818622

**Published:** 2026-08-27

**Authors:** Haakon Solum, Zoe Stephenson

**Affiliations:** School of Psychology, University of Birmingham, Birmingham, United Kingdom

**Keywords:** determinism, dualism, free will beliefs, Free Will Inventory, reliability, validity

## Abstract

This review critically evaluates the Free Will Inventory (FWI), a 29-item self-report instrument assessing beliefs about free will, determinism, and dualism, by situating it within broader philosophical and psychological frameworks and prior attempts to measure free will beliefs. To identify relevant peer-reviewed studies, a structured literature search using systematic principles was conducted to locate research that has validated or critiqued the FWI. The review also considers how free will belief is conceptualised and operationalised within the FWI, and the implications this has for interpreting its scores. The reviewed evidence indicates generally acceptable internal reliability across multiple international samples, although no published studies have assessed test–retest reliability. Existing findings support aspects of construct, face, and content validity, while criterion validity remains largely unexplored, and several methodological considerations - including the potential influence of social desirability, demand characteristics, and cultural context - have been noted. Overall, the literature suggests that the FWI is a conceptually grounded and moderately reliable measure of free will beliefs, but further research is needed to clarify its temporal stability, cross-cultural robustness, practical applications, and the conceptual interpretation of its scores.

## 1 Introduction

The debate over the existence of free will is an ancient one. As early as 350 BCE, Aristotle considered that individuals had the power “to do or not to do” - speaking to a sense of human agency and free choice. Many in the modern-day seem to identify with Aristotle’s sentiments; the belief in free will has been found to be high in the general population (Nahmias et al., 2005). Some scholars argue that a strong belief in free will is a helpful evolutionary adaptation, as a higher belief in free will has been found to have prosocial benefits such as reducing aggression and increasing helpfulness (Baumeister et al., 2009). Such beliefs also serve to assuage the guilt that emerges when seeking to punish those who transgress against the group (Clark et al., 2017). Relatedly, a belief in free will and that people are morally responsible for their actions also underpins the philosophical framework of many countries’ Criminal Justice Systems (Greene and Cohen, 2004). If a person’s free will is considered to be compromised - for example, due to mental illness – they may be exculpated on the grounds that they are not fully responsible for their actions (Meynen, 2016).

Determinism is a philosophical position that posits that all events (including human actions) are causally predetermined by prior causes (Harris, 2012), a position which some believe makes it impossible for people to have free will (Fischer et al., 2007). The philosophical position that free will and determinism are mutually exclusive is called incompatibilism (Kane, 1998). Incompatibilists argue that a deterministic universe would make it impossible for people to truly have free will and make choices that are not predetermined. An alternative philosophical position, compatibilism (Fischer et al., 2007), stipulates that free will can exist in a deterministic universe, i.e., people can still have free will even if they are affected by external influences and if given the same choice again, individuals could choose differently. Some compatibilists argue that while we may live in a deterministic universe, free will is still possible if we can freely identify with some of our desires over others (Frankfurt, 1988). Others suggest that free will is the capacity to make choices based on our ability to reason, which can be considered separate from the physical world [e.g., Kant, 2006 or mind–body dualism (Bracken and Thomas, 2002)]. In other words, free will beliefs are not binary, and there are multiple nuanced positions on the topic of free will.

Importantly, beliefs about free will are not merely abstract philosophical positions. They shape real-world attitudes and behaviours in various areas such as criminal justice, education and interpersonal relationships. For instance, people who endorse higher beliefs in free will are more likely to endorse punitive punishment than those who doubt it (Shariff et al., 2014).

Researchers have developed psychometric measures to assess beliefs about free will. Evaluating the reliability and validity of these tools is essential to ensure the accuracy of research findings, support replicability, contribute to theoretical development, and enable meaningful applications across different contexts. One promising tool is the Free Will Inventory (FWI: Nadelhoffer et al., 2014). As such, this article will provide a comprehensive critique of the FWI. To do this, initial reference will be made to some commonly used measures of free will. This will be followed by an overview of the FWI and a discussion of the reliability and validity of the measure. The article will conclude with a consideration of the use of the FWI in research.

Since the 1980s, different tools have been created to measure people’s beliefs in free will (e.g., Paulhus and Carey, 2011; Paulhus and Margesson, 1994; Rakos et al., 2008; Stroessner and Green, 1990; Viney et al., 1988; Viney, 1982). However, many of these tools exhibit identifiable issues regarding their reliability and validity. For example, one of the first free will measures, the Free Will and Determinism scale (FWD: Viney et al., 1988), has an inbuilt incompatibilist view of free will such that it operationally defines free will in opposition to determinism. Consequently, this measure may be limited in capturing compatibilist views despite compatibilism being the prevailing stance among philosophers in the free will debate (Bourget and Chalmers, 2014) and the general population (Monroe and Malle, 2010).

A more recent scale, also named the Free Will and Determinism Scale (Rakos et al., 2008), incorporated the same incompatibilist assumptions. Further, it was validated with fewer than 100 participants, limiting the robustness of its findings. The Free Will and Determinism Plus (FAD-Plus) (Paulhus and Carey, 2011) is a newer and widely used instrument in free will research (Crone and Levy, 2019). However, the FAD-Plus does not include a dualism scale, thereby not allowing for understanding people’s beliefs about dualism in relation to free will. Finally, the FAD-Plus is thought to conflate belief in free will with moral responsibility with items such as “People must take full responsibility for any bad choices they make” and “Criminals are totally responsible for the bad things they do” (Nadelhoffer et al., 2014).

Accurately capturing individual differences in belief in free will is essential given the philosophical and societal implications of these beliefs. Empirical studies have linked belief in free will to a range of psychological and behavioural outcomes, including moral judgment (Baumeister et al., 2009), willingness to punish (Shariff et al., 2014), and even levels of aggression (Baumeister et al., 2009). These findings highlight the need for robust and valid tools to measure free will beliefs reliably across contexts. In response to this need, the Free Will Inventory (FWI; Nadelhoffer et al., 2014) was developed with the aim of improving upon earlier measures. The authors’ stated intention was to create a tool with greater conceptual validity, aligning more closely with how key terms in the free will debate have traditionally been understood by philosophers (Nadelhoffer et al., 2014).

In developing the Free Will Inventory, Nadelhoffer et al. (2014) began by generating a broad set of items designed to capture the main philosophical positions on free will, determinism, and dualism. Items were drawn from existing scales, conceptual analyses, and philosophical literature to ensure comprehensive coverage of the constructs. Across four rounds of validation with over 1,100 U.S. participants, the authors refined item wording and structure, informed by conceptual guidance from philosophy and psychology literature, alongside empirical data. Confirmatory factor analysis was used to test whether the items clustered according to the intended subscales, and poorly performing items were revised or removed. Part 1 items were designed to measure the strength of belief in each construct, while Part 2 items were created to explore the relationships between beliefs about free will, moral responsibility, punishment, and dualism, allowing for finer-grained investigation of participants’ philosophical intuitions.

The FWI is a two-part, 29-item self-report questionnaire that is designed to measure individuals’ beliefs and attitudes towards free will and related concepts. The first part attempts to measure the strength of a person’s belief in free will, determinism and dualism (three subscales). Part two examines the relationship between these views. It is considered easy to administer, and participants do not require prior knowledge beyond reading skills. Participants are asked to indicate, on a 7-point Likert scale, their agreement or disagreement with a range of statements.

The manual accompanying the FWI provides instructions on how to administer and interpret the responses from participants. Higher scores on each subscale indicate stronger endorsement of the underlying construct.

The authors of the FWI argued that previous free will measures (e.g., Paulhus and Carey, 2011; Viney, 1982) failed to adequately capture the main philosophical positions in the debate, which they viewed as a limitation. The authors stated that the FWI was developed to measure free will beliefs in a way that aligns with the main philosophical positions on the topic.

The three sub-scales in the FWI are “Free Will,” “Determinism,” and “Dualism.” The Free Will sub scale investigates the strength of belief in the existence of free will. The Determinism subscale measures a person’s degree of belief in determinism (i.e., the idea that events are influenced by prior events). The Dualism sub-scale measures a person’s strength of belief in mind–body dualism. Including a dualism sub-scale, whilst allowing for the exploration of individuals’ views on incompatibilist and compatibilist ideas, represents a novel addition to free will measurement (Nadelhoffer et al., 2014) (See Table 1 for item statements and descriptions).

**Table 1:** Items of the Free Will Inventory (FWI).

Part 1 (by subscale)			
Subscale	Item no	Statement	Description of domain
Free Will (FW)	1	People always have the ability to do otherwise.	Captures beliefs that humans can make choices independent of prior causes, external constraints, or deterministic laws. Emphasizes personal control and alternative possibilities.
Free Will (FW)	2	People always have free will.	
Free Will (FW)	3	How people’s lives unfold is completely up to them.	
Free Will (FW)	4	People ultimately have complete control over their decisions and their actions.	
Free Will (FW)	5	People have free will even when their choices are completely limited by external circumstances.	
Determinism (DE)	1	Everything that has ever happened had to happen precisely as it did, given what happened before.	Captures beliefs that all events, including human choices, are fully determined by prior causes, laws of nature, or initial conditions, leaving no room for genuine alternatives.
Determinism (DE)	2	Every event that has ever occurred, including human decisions and actions, was completely determined by prior events.	
Determinism (DE)	3	People’s choices and actions must happen precisely the way they do because of the laws of nature and the way things were in the distant past.	
Determinism (DE)	4	A supercomputer that could know everything about the way the universe is now could know everything about the way the universe will be in the future.	
Determinism (DE)	5	Given the way things were at the Big Bang, there is only one way for everything to happen in the universe after that.	
Dualism/Anti-Reductionism (DU)	1	The fact that we have souls that are distinct from our material bodies is what makes humans unique.	Captures beliefs that human minds or souls are distinct from physical processes and cannot be fully explained by neuroscience or biology alone.
Dualism/Anti-Reductionism (DU)	2	Each person has a non-physical essence that makes that person unique.	
Dualism/Anti-Reductionism (DU)	3	The human mind cannot simply be reduced to the brain.	
Dualism/Anti-Reductionism (DU)	4	The human mind is more than just a complicated biological machine.	
Dualism/Anti-Reductionism (DU)	5	Human action can only be understood in terms of our souls and minds and not just in terms of our brains.	

Part 2: measuring relationships between beliefs		
Item no	Statement	Description
1	Free will is the ability to make different choices even if everything leading up to one’s choice (e.g., the past, the situation, and desires, beliefs, etc.) were exactly the same.	Part 2 is designed to explore how individuals’ beliefs about free will relate to their views on moral responsibility, punishment, determinism, and the nature of the mind or soul. The items examine different philosophical positions, including libertarian and compatibilist conceptions of free will, as well as attitudes toward retributive and rehabilitative approaches to punishment. They also probe beliefs about the existence of non-physical aspects of the self and the predictability of human behaviour, offering a more nuanced picture of participants’ underlying intuitions and reasoning about these complex issues.
2	To be responsible for our present decisions and actions we must also be responsible for all of our prior decisions and actions that led up to the present moment.	
3	Free will is the ability to make a choice based on one’s beliefs and desires such that, if one had different beliefs or desires, one’s choice would have been different as well.	
4	People deserve to be blamed and punished for bad actions only if they acted of their own free will.	
5	People could have free will even if scientists discovered all of the laws that govern all human behaviour.	
6	People who harm others deserve to be punished even if punishing them will not produce any positive benefits to either the offender or society (e.g., rehabilitation, deterring other would-be offenders).	
7	To have free will means that a person’s decisions and actions could not be perfectly predicted by someone else no matter how much information they had.	
8	People who perform harmful actions ought to be rehabilitated so they no longer pose a threat to society.	
9	If it turned out that people lacked non-physical (or immaterial) souls, then they would lack free will.	
10	People who perform harmful actions ought to be punished so that other potential offenders are deterred from committing similar harmful actions.	
11	To have free will is to be able to cause things to happen in the world without at the same time being caused to make those things happen.	
12	People could be morally responsible even if scientists discovered all of the laws that govern human behaviour.	
13	People have free will as long as they are able to do what they want without being coerced or constrained by other people.	
14	If it turned out that people lacked non-physical (or immaterial) souls, then they would lack moral responsibility.	

Adapted from Nadelhoffer et al. (2014, p. 34–35, 38). Response options for all items range from 1 (strongly disagree) to 7 (strongly agree).

As previously outlined, the authors of the FWI (Nadelhoffer et al., 2014) argued that the pre-existing free will measures were problematic and in need of improvement. Through four rounds of validation using a sample of 1,163 US participants, the authors developed and validated Part One of the FWI. One of their reported goals was to maximise construct validity. The authors of the FWI stated that they aimed to use a more representative US sample, addressing a key limitation of previous validation efforts. For example, the FAD-Plus was validated primarily with white college students and U.S. residents of European heritage (Nadelhoffer et al., 2014). For instance, one round (round 3) of the FWI validation included 317 participants with a gender distribution of 48% male and 51% female. The sample was more ethnically diverse [the FAD-Plus sample did not include minority participants (Paulhus and Carey, 2011), with 86% identifying as White/Caucasian, 7% as Black/African-American, 4% as Asian/Pacific Islander, 3% as Hispanic/Latino, and 1% as Native American]. The ages of participants ranged from 20 to 80, with a mean age of 49.3 years (SD = 13.07). They used confirmatory factor analysis to validate Part One of the FWI and to show that the sub-scales measured independent concepts.

The FWI is divided into two parts, which can be administered separately or together depending on research aims. Part One measures the *strength* of a person’s beliefs in three constructs (i.e., free will, determinism, and dualism/non-reductionism) using three corresponding five-item subscales. Higher cumulative scores on each subscale indicate stronger endorsement of that construct. Part Two examines the *relationships* between these beliefs and related concepts such as moral responsibility and punishment. It consists of items reflecting different philosophical positions (e.g., libertarianism, compatibilism, retributivism, dualism) and the perceived implications of holding particular beliefs about free will and determinism. Unlike Part One, Part Two is not scored cumulatively; instead, responses are typically analysed at the item level to explore patterns in how participants connect or distinguish these concepts (See Table 1 for Part 2 item statements).

The FWI employs ordinal data as its level of measurement, allowing responses to be ranked without assuming equal intervals between response options. Participants are asked to use a 7-point Likert scale to rate their agreement or disagreement with different statements, ranging from “strongly disagree” to “strongly agree.” Ordinal scales do not allow for precise measurement of the intervals between response categories. However, they do enable researchers to identify the direction of responses and compare them across participants. The use of ordinal scales is common in psychological research (Schnuerch et al., 2022). In the context of free will research, measuring beliefs using interval or ratio scales would be challenging, as such beliefs are inherently subjective. Therefore, researchers employing the FWI must carefully select appropriate statistical methods for analysing ordinal data.

The FWI is a self-report measure which can be completed without any prior training or explanation. This makes it a cost-effective measure for projects with limited funding or large sample sizes. In free will research, all prior measures (to the authors’ knowledge) have also relied on self-report, as it is perceived that this is likely the most effective way of accessing participants’ personal beliefs.

As with all self-report measures, consideration must be given to different types of potential bias. Regarding the FWI, it is unlikely that social desirability bias will be a significant problem. Most participants are unlikely to view their beliefs in free will as a particularly important social position. However, in some research conditions that, for example, attempt to investigate free will beliefs in scenarios revolving around questions of punishment or similar topics (Shariff et al., 2014), it is possible to imagine that participants could alter their responses to be perceived in a more favourable light. Similarly, demand characteristics could bias some participants in specific research scenarios. In Part One of the FWI, it is perhaps unlikely that these biases would significantly impact responses as the questions could be considered potentially benign. For instance, a question such as “Every event that has ever occurred, including human decisions and actions, was completely determined by prior events” does not appear to invite socially desirable responses. However, Part Two includes statements such as, “People who harm others deserve to be punished even if punishing them will not produce any positive benefits to either the offender or society, e.g., rehabilitation, deterring other would-be offenders, etc.” Responses to such items may be influenced by factors such as culture, political orientation, or social desirability, potentially leading individuals to over- or under-report their punitive attitudes.

Brenner and DeLamater (2014) argued that social desirability bias could exist in online surveys because people may answer questions to define their identities. Being “tough on crime” might seem appropriate in some circles (Newburn, 2007). In contrast, holding rehabilitation-focused beliefs might seem appropriate in others. However, due to most researchers ensuring participant anonymity in their research, it is suggested that participants will be less prone to social desirability bias (Dodou and de Winter, 2014).

These considerations point to a more fundamental question underlying any evaluation of the FWI: what exactly is being measured when researchers assess “belief in free will”? This question is important because free will belief can be understood in several different ways, each of which has different implications for measurement. Belief in free will has been characterised in terms of alternative possibilities, sourcehood, self-control, rational deliberation, agency attribution, moral responsibility, and attitudes towards blame and punishment (e.g., Feldman, 2017; Nadelhoffer et al., 2014). Feldman (2017), for example, distinguishes belief in free will from related agency constructs such as self-control, autonomy, locus of control, and intentionality, arguing that it captures a distinct aspect of perceived agency. These dimensions need not coincide. A person may believe that individuals can choose and act without constraint while remaining agnostic about indeterminism, or may endorse moral responsibility without holding a specific metaphysical view of causation. Whether the three constructs targeted by the FWI (i.e., free will, determinism, and dualism) adequately capture this broader space of belief is therefore an open question.

A related concern is whether the FWI’s constructs map onto how ordinary people understand free will. The FWI was deliberately designed to align with the vocabulary of philosophical debate, including philosophical definitions of determinism and dualism (Nadelhoffer et al., 2014). However, empirical work suggests that lay concepts of free will may be primarily psychological rather than metaphysical. When asked to define free will, people commonly refer to the capacity to make choices in line with their desires and to act free from coercion or constraint, with less spontaneous reference to indeterminism or the soul (Feldman, 2017; Monroe and Malle, 2010). Similarly, Monroe et al. (2014) found that the perceived capacity for choice, rather than metaphysical commitments such as belief in souls, predicted both free-will and blame judgements. If lay belief is centred primarily on choice and constraint, then the FWI’s Determinism and Dualism subscales may capture dimensions that are salient within philosophical debate but comparatively peripheral to ordinary free-will belief.

Nadelhoffer et al. (2014) were themselves alert to aspects of this issue. They note that Part One measures the strength of belief in free will, determinism, and dualism, rather than the specific content of those beliefs. For example, a compatibilist and an incompatibilist could endorse the same free-will items while understanding free will differently. Part Two was therefore developed to explore the relationships among beliefs about choice, responsibility, the soul, punishment, and related concepts. However, this also raises an interpretive issue. These items span several kinds of belief, including metaphysical claims about indeterminism and immaterial minds, psychological claims about choice and control, moral claims about responsibility and desert, and socially relevant attitudes towards punishment. These need not reflect the same underlying disposition. Responses to punishment items, in particular, may reflect moral values, religiosity, political orientation, or cultural context as much as belief about free will narrowly understood. Clarifying which level of belief the FWI is best understood to measure is therefore an important precondition for interpreting its psychometric properties, and this issue is returned to below in the discussion of construct validity.

These issues suggest that a critical review of the FWI cannot focus solely on conventional psychometric criteria, but must also consider the conceptual clarity of the construct being measured. Given the conceptual improvements and broader applicability of the FWI, it is therefore important to consider both how the measure operationalises belief in free will and the extent to which it meets accepted psychometric standards. While several studies have used the FWI, there remains a lack of consolidated, critical evaluation of its reliability, validity, and performance across different populations and contexts, highlighting a clear research gap. Accordingly, the objective of this review is twofold: first, to clarify what is being measured when researchers assess “belief in free will” (that is, how the construct is operationalised and whether its dimensions map onto lay belief), and second, to synthesise and critically evaluate all available empirical evidence on the psychometric properties of the FWI, including reliability, multiple forms of validity, and cross-contextual applicability. Because the validity of any instrument depends on the clarity of the construct it targets, these two aims are closely linked. The review adopts a structured narrative approach, employing systematic search principles to ensure transparency and reproducibility in study identification, while allowing for the interpretive and critical analysis that characterises narrative synthesis. The following sections provide a structured examination of the FWI across study populations, cultural contexts, and measurement domains.

## 2 Method

A structured literature search was conducted across Web of Science, PsycInfo, and Scopus to identify peer-reviewed studies reporting on the Free Will Inventory (FWI; Nadelhoffer et al., 2014). The search was performed using the following Boolean search string in each database:

“Free Will Inventory” OR “FWI.”

AND

“psychometric” OR “reliability” OR “validity” OR “factor analysis” OR “confirmatory factor analysis” OR “scale development” OR “internal consistency” OR “Cronbach’s alpha.”

Searches were limited to publications from 1 January 2014 (the year the FWI was first published) to 15 December 2025. Only studies written in English and reporting empirical data on the FWI’s psychometric properties were included; commentary, opinion pieces, or purely theoretical papers were excluded.

**Figure 1:**
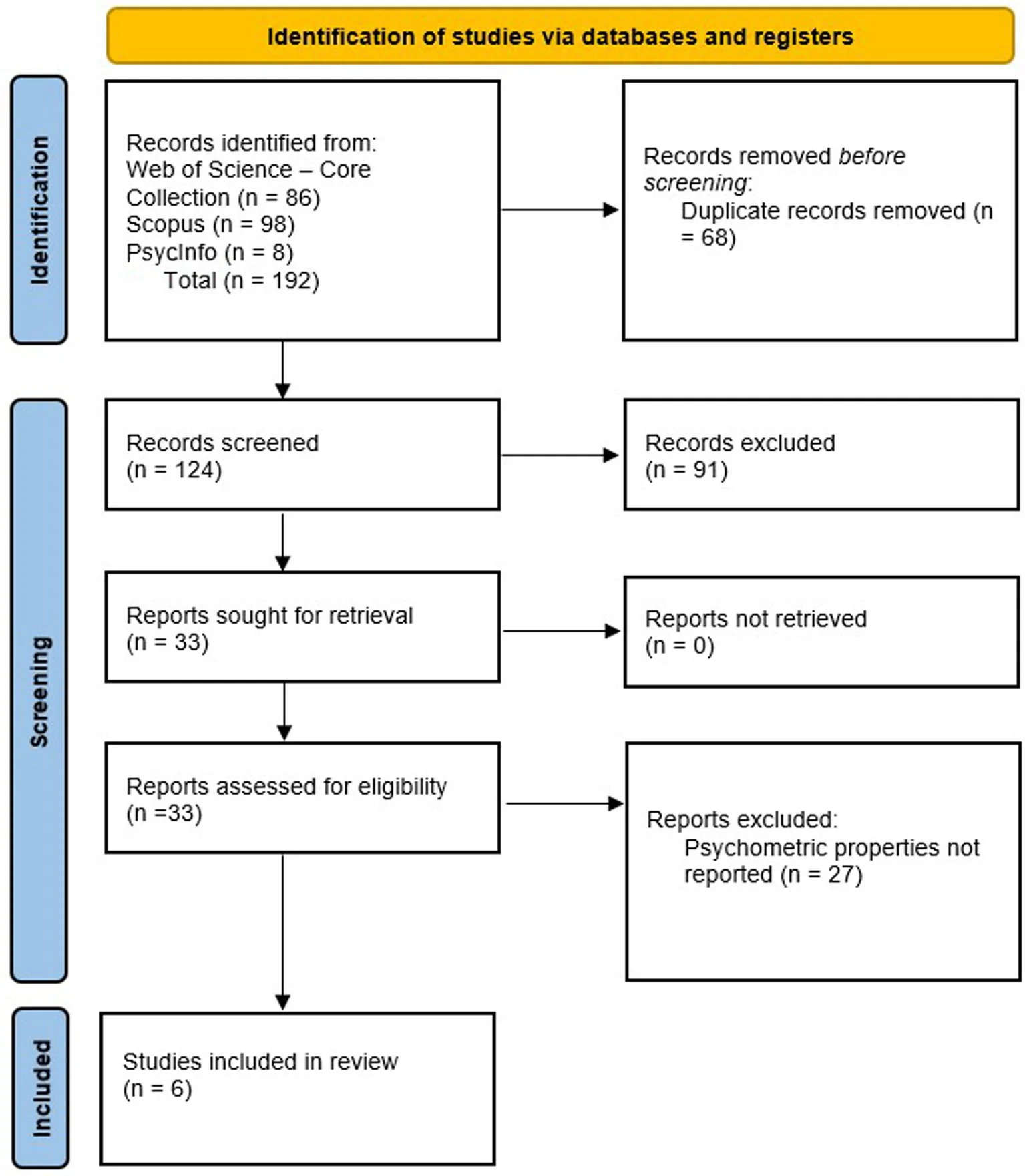
PRISMA flowchart (adapted from Page et al., 2021).

Following the searches, duplicate records were removed across the three databases. Titles and abstracts were screened against inclusion criteria, and potentially relevant studies were retrieved for full-text review. The selection process is summarised in Figure 1 (PRISMA flow diagram).

For each included study, information was extracted on authors, year, sample characteristics, and the specific psychometric properties evaluated (e.g., internal consistency, test–retest reliability, content validity, construct validity, convergent validity, discriminant validity, predictive validity). Where reported, additional details such as cross-cultural adaptations, translation procedures, and methodological considerations were also recorded. Extracted data are summarized in Table 2, which indicates which authors assessed which aspects of reliability and validity; all numerical results and statistical analyses are presented in the Results section.

**Table 2:** Summary of psychometric properties of the Free Will Inventory (FWI).

Author(s)	Sample/country	Type of FWI version	Key findings
Crone and Levy (2019)	Total *N* = 921 (4 studies: 210, 220, 294, 197) United States	Original	Internal consistency Study 1: *α* = 0.88 Study 2: *α* = 0.89 Study 3: *α* = 0.85 Concurrent validity FWI and FAD-PLUS were strongly correlated with each other (*r*s = 0.85, 0.82, and 0.76) across Studies 1–3.
Genschow et al. (2022)	Total *N* = 250 Germany	Translated	Internal consistency Free Will subscale *α* = 0.59 Determinism *α* = 0.76 Dualism *α* = 0.75
Nadelhoffer et al. (2014)	*n* = 293, 321, 317, 232 (across 4 EFA rounds), 330 (CFA) United States	Original	Internal consistency Free Will subscale *α* = 0.80 Determinism α = 0.77 Dualism *α* = 0.77 Confirmatory factor analysis 15-item final scale; fit indices acceptable (GFI = 0.927, CFI = 0.931, AGFI = 0.900, RMSEA = 0.058); standardized regression weights confirmed items loaded appropriately on intended factors Discriminant validity FW moderately correlated with DU, weakly with DE, supporting distinct but related constructs.
Peng et al. (2024)	Total *N* = 1,028 (across 3 experiments) Belgium	Translated	Internal consistency Free Will (FW): *α* = 0.76 Determinism (DE): *α* = 0.77 Dualism (DU): *α* = 0.82 Convergent validity (DU subscale) with RPBS (paranormal beliefs): *r* = 0.58 DU correlations with false alarm rate in pattern detection tasks: *r* = 0.13–0.16 Mediation: after controlling for paranormal beliefs, DU, false alarm rate is non-significant
Vilanova et al. (2018)	Total *N* = 316 Brazil	Translated	Internal consistency Free Will (FW): *α* = 0.81 Determinism (DE): *α* = 0.75 Dualism (DU): *α* = 0.85
Wisniewski et al. (2019)	Total *N* = 1,900 USA (*n* = 900) & Singapore (*n* = 900) US & Singapore	Original	Internal consistency - US Sample Free Will (FW): *α* = 0.81 Determinism (DE): *α* = 0.85 Dualism (DU): *α* = 0.82 Internal consistency - Singapore Sample Free Will (FW): *α* = 0.79 Determinism (DE): *α* = 0.81 Dualism (DU): *α* = 0.78

A narrative synthesis approach was adopted, structured according to the key psychometric properties of the FWI. Where directly relevant empirical studies were unavailable, discussion and interpretation drew on broader psychometric, methodological and conceptual literature to provide context and highlight implications for scale development, evaluation, and use.

The structured search was designed to identify empirical studies of the FWI’s psychometric properties; the review’s conceptual aim, concerning how the FWI operationalises belief in free will, was addressed through engagement with relevant theoretical and empirical literature on free-will belief.

## 3 Results

The following sections present the available psychometric evidence on the Free Will Inventory (FWI) as a narrative summary. Within each sub-section, descriptive findings are reported first, with interpretive commentary included to highlight patterns, contextual considerations, or implications. This commentary may arise from the statistical results themselves or from theoretical and comparative considerations where no formal statistics are available (e.g., test–retest reliability).

### 3.1 Reliability

Broadly speaking, reliability refers to the consistency of a psychometric instrument - whether it yields stable and dependable results across time, items, and contexts (Kline et al., 2000). A reliable measure ensures that any observed variation in responses is due to actual differences in the construct being measured, rather than inconsistencies in the tool itself. In the context of free will belief research, reliability is important given the abstract and potentially fluctuating nature of such beliefs. This section reviews the available literature on the reliability of the Free Will Inventory (FWI), focusing specifically on internal consistency (i.e., how consistently the items within each subscale measure the same construct) and test–retest reliability (i.e., the stability of scores over time).

#### 3.1.1 Internal consistency

Internal consistency evaluates the degree to which items within a subscale consistently measure the same underlying construct (Kline et al., 2000). The FWI demonstrates satisfactory results in this regard. The internal consistency (i.e., the degree to which the items within a questionnaire or test measure the same underlying construct) of the FWI is robust, with the five-item Free Will sub-scale yielding a Cronbach’s *α* of 0.80, the five-item Determinism subscale yielding Cronbach’s α = 0.77, and the five-item dualism subscale yielding Cronbach’s *α* = 0.77 (*n* = 330, Nadelhoffer et al., 2014). In addition, Crone and Levy (2019) reported high internal consistency for both the FWI and FAD-Plus subscales across four U.S. studies (αs ranging from 0.85 to 0.90), further supporting the reliability of the FWI in large, adult samples. The FWI has been used in other countries and notably translated into other local languages, resulting in similar results. However, in a study conducted in Germany (Genschow et al., 2021), the translated German version showed substantially lower internal consistency for the Free Will subscale (α = 0.59) compared with the Determinism (α = 0.76) and Dualism (α = 0.75) subscales (*n* = 250).

In Brazil, Vilanova et al. (2018) reported good internal reliability, with Cronbach’s α values of 0.81 for the Free Will subscale, 0.75 for Determinism, and 0.85 for Dualism (*n* = 316). Similarly, Wisniewski et al. (2019) examined representative samples from the United States and Singapore. For the U.S. sample (*n* = 900), internal reliability was α = 0.81 for Free Will, α = 0.85 for Determinism, and α = 0.82 for Dualism. For the Singaporean sample (*n* = 900), the corresponding values were α = 0.79, α = 0.81, and α = 0.78, respectively. More recently, Peng et al. (2024) reported internal consistency for the Free Will Inventory (FWI) subscales in a Chinese sample, with Cronbach’s alpha values of 0.76 for Free Will, 0.77 for Determinism, and 0.82 for Dualism. These results support the FWI’s internal reliability across cultural contexts, though the study did not assess its validity or broader psychometric structure.

Cronbach’s alpha scores suggest that, overall, the FWI has a moderate to high level of internal consistency (Kline et al., 2000) and, as such, can be considered acceptable for research purposes. However, in the study by Genschow et al. (2021), the score for free will fell below 0.60, indicating that the German translation potentially measures this concept inconsistently. These findings indicate that while the FWI generally demonstrates acceptable internal consistency across diverse cultural contexts, translated versions may introduce variability in how subscales function.

Lastly, the brevity of the FWI subscales (each containing only five items) enhances practicality but may limit internal consistency, particularly in translated versions where subtle shifts in item meaning can have greater proportional impact (Rammstedt and Beierlein, 2014).

Taken together, the available evidence suggests that the Free Will Inventory demonstrates generally acceptable internal consistency across multiple cultural contexts, particularly for the Determinism and Dualism subscales. However, the notably reduced internal reliability of the Free Will subscale in the German translation raises concerns about the stability and conceptual clarity of this construct across languages. This variability highlights the sensitivity of short subscales to translation effects and suggests that internal consistency cannot be assumed to generalise across cultural contexts without empirical verification. While the FWI’s brevity enhances its practicality and appeal for large-scale research, it may also exacerbate reliability limitations in translated versions, underscoring the need for careful cross-cultural validation and the use of complementary reliability indices beyond Cronbach’s alpha.

#### 3.1.2 Test re-test reliability

Test re-test reliability refers to whether the results of a test will remain similar over time and if the results can be replicated with the same subjects (Kline et al., 2000). To date, no published studies have formally assessed the test–retest reliability of the Free Will Inventory (FWI), representing a gap in the evaluation of its temporal consistency and score stability. However, discussing this aspect remains important, as it raises broader questions about whether free will beliefs are best understood as stable traits or as more fluid, context-dependent states. This distinction is relevant because it shapes expectations about temporal consistency. For example, the State Anxiety scale of the State–Trait Anxiety Inventory (STAI) demonstrates lower test–retest reliability than the Trait Anxiety scale of the same measure (Vitasari et al., 2011). This difference reflects the transient nature of state measures and the stability of trait measures. If free will beliefs are viewed as a state indicator, it follows that an individual’s beliefs may vary over time, influenced by situational circumstances or other factors. For example, research has shown that the strength of free will beliefs can be sensitive to effective manipulations (e.g., Nichols, 2006). Research suggests that psychoeducational material highlighting biopsychosocial influences on behaviour can reduce free will beliefs (Katz and Fondacaro, 2021). Baumeister et al. (2009) induced disbelief in free will by asking participants to read passages promoting determinism.

The FWI has been used and validated in different countries and across cultures (Genschow et al., 2021; Nadelhoffer et al., 2014; Vilanova et al., 2018), which suggests that it is capturing something that is fundamental regarding individuals’ beliefs in free will. The existence of constructs across cultures does not necessarily imply that the FWI is capturing a stable trait. It is possible that the construct of free will beliefs may have universal elements and core ideas shared across cultures. However, this may indicate that the FWI captures the presence of these beliefs rather than their stability over time. If this is the case, the test re-test reliability of the FWI should be expected to be lower due to the naturally occurring fluctuations in someone’s free will beliefs. Although the authors of the FWI do not specify whether they view free will beliefs as stable traits or momentary states, beliefs are generally considered relatively stable constructs (Barnby et al., 2019). Many researchers (e.g., Paulhus and Carey, 2011) describe free will beliefs as enduring traits rather than states. This could suggest that free will beliefs (at least theoretically) should remain stable across time and contexts. In light of this theoretical ambiguity, future research is needed to determine whether the FWI captures enduring beliefs or momentary attitudes, and to establish its test–retest reliability accordingly.

In summary, the absence of published test–retest reliability data represents a significant gap in the psychometric evaluation of the Free Will Inventory. While theoretical arguments can be made for understanding free will beliefs as either relatively stable traits or context-sensitive attitudes, the lack of empirical evidence means that the temporal stability of FWI scores remains unknown. This limits confidence in the use of the FWI for longitudinal research, intervention studies, or designs that assume stability of beliefs over time. Until test–retest reliability is empirically established, researchers should exercise caution when interpreting changes in FWI scores as meaningful shifts in belief rather than potential measurement variability or situational effects.

### 3.2 Validity

Validity refers to the extent to which a psychometric instrument measures what it intends to measure. Evaluating the validity of the Free Will Inventory (FWI) is essential to ensure that findings based on the tool can be interpreted meaningfully and applied appropriately across research settings. This section reviews the literature on the FWI’s face, concurrent, content, construct, and predictive validity.

#### 3.2.1 Face validity

Unlike other forms of validity, face validity is a qualitative judgement concerning whether an instrument’s items appear, at face value, to measure their intended constructs (Holden, 2010; Kline et al., 2000). This judgement typically considers factors such as clarity, relevance, and ease of comprehension. In the case of the Free Will Inventory, some items - particularly in Part Two - may be challenging for lay respondents to interpret due to the complexity of the language and underlying philosophical concepts. For example, statements such as “Free will is the ability to make a choice based on one’s beliefs and desires such that, if one had different beliefs or desires, one’s choice would have been different as well” (Nadelhoffer et al., 2014) require engagement with abstract philosophical distinctions. While such complexity may be difficult to avoid when assessing nuanced metaphysical beliefs, similar challenges have been noted in the development of other free will measures, including the FAD-Plus (Paulhus and Carey, 2011). In response to these issues, the authors of the FWI undertook multiple rounds of item refinement during scale development, in part to improve face validity (Nadelhoffer et al., 2014).

In summary, the face validity of the Free Will Inventory appears mixed. Items in Part One generally align clearly with their intended constructs and are likely to be accessible to most respondents, whereas some Part Two items rely on philosophically dense formulations that may hinder comprehension among general population samples. Consequently, while the FWI’s face validity may be adequate for use with philosophically informed participants, it may be more variable in broader samples. Researchers should therefore consider participant background and research aims when deciding whether to include Part Two items and when interpreting responses to conceptually complex statements.

#### 3.2.2 Concurrent validity

Concurrent validity refers to the extent to which an instrument correlates with another measure of the same construct administered at the same time (Campbell and Fiske, 1959). In free will research, the Free Will Inventory has most commonly been examined in relation to the FAD-Plus (Paulhus and Carey, 2011), another widely used measure with established internal consistency. Evidence from three studies indicates strong positive correlations between the FWI and the FAD-Plus (*r* = 0.85, 0.82, and 0.76; Crone and Levy, 2019), supporting the concurrent validity of the FWI. However, because this evidence is based on a single comparator, further validation against a broader range of free will measures and related constructs would strengthen confidence in the scope and robustness of the FWI’s concurrent validity.

#### 3.2.3 Convergent and discriminant validity

Convergent validity, a subtype of construct validity, refers to the extent to which a measure correlates with other theoretically related instruments, whereas discriminant validity assesses whether the measure is sufficiently distinct from related but conceptually different constructs (Campbell and Fiske, 1959).

Evidence for the FWI’s convergent validity comes from correlations with the FAD-Plus (Paulhus and Carey, 2011), a widely used measure of free will beliefs. Crone and Levy (2019) report strong positive correlations between the FWI and FAD-Plus (*r* = 0.85, 0.82, and 0.76 across three studies), supporting the conclusion that the FWI effectively captures the intended construct.

More recently, Peng et al. (2024) demonstrated that the Dualism subscale of the FWI correlates positively with paranormal beliefs (RPBS; *r* = 0.58) and with false alarm rates in pattern detection tasks, providing additional support for the FWI’s convergent validity in relation to theoretically related constructs. Importantly, when controlling for paranormal beliefs, the direct relationship between Dualism and false alarm rates was no longer significant, indicating that the association with perceptual outcomes is mediated by a related construct rather than reflecting a direct effect of Dualism itself. This pattern aligns with expectations for convergent validity, showing that Dualism relates meaningfully to conceptually connected constructs while highlighting the role of mediating variables in downstream behavioural measures.

Discriminant validity can be evaluated via correlations among the FWI subscales. The Free Will subscale shows moderate correlation with Dualism and weak correlation with Determinism (Nadelhoffer et al., 2014), suggesting that while related constructs are partially associated, they largely remain distinct. The observed overlap, particularly between Free Will and Dualism, likely reflects conceptual proximity rather than measurement error (Nadelhoffer et al., 2014).

Overall, these findings provide preliminary support for the convergent and discriminant validity of the FWI. However, the evidence is limited: convergent validity relies on a single comparator, and discriminant validity is assessed via simple correlation matrices rather than standardized approaches such as average variance extracted (AVE) or heterotrait–monotrait (HTMT) ratios. Future research could strengthen this evidence by applying formal, standardized tests across multiple independent measures.

#### 3.2.4 Content validity

Content validity refers to whether an instrument encompasses the entire content domain related to the construct (Sireci, 2007). If a measure falls short in this area, it will not capture the complete breadth of the domain under assessment. As the introduction outlines, the FWI is seemingly the most comprehensive among existing measures encompassing the principal philosophical positions on free will beliefs. Of particular significance is the FWI’s incorporation of aspects pertaining to dualism. Some research (e.g., Wisniewski et al., 2019) purports that free will beliefs are better predicted by beliefs in dualism than determinism and thus indicates the importance of including this aspect. Whilst the authors assert that the FWI reflects the major philosophical positions, they acknowledge that it does not capture all possible nuances in individual beliefs about free will (Nadelhoffer et al., 2014).

Item development and refinement were guided by the authors’ expertise in philosophy and psychology, who evaluated items for conceptual relevance, alignment with the intended construct, and clarity of language. Items were revised iteratively based on these expert judgments alongside empirical evidence from four rounds of exploratory factor analysis (EFA), which served as the primary criterion for retaining, revising, or dropping items. It is worth noting that this iterative EFA-driven process primarily speaks to construct validity - that is, whether items cohere empirically around their intended factors - rather than to content validity in the formal psychometric sense. Procedures such as independent expert panels or quantitative agreement indices (e.g., CVI, CVR) are not reported, which reflects a broader pattern in scale development literature of this period rather than an idiosyncratic limitation of the FWI specifically. Future validation work might usefully address this by applying more formalised content validation procedures.

The three factors identified through the validation process might meet the authors’ goals of constructing a measure that covers the main philosophical positions on the free will topic. However, as outlined in the section below, there were some difficulties with disentangling the distinct concepts during the factor analysis. While some challenges were addressed (e.g., wording items differently after the first round to address the overlap between factors and ambiguity), the last round of factor analysis resulted in limited loading of items beyond three factors. A fifth round of data collection employed confirmatory factor analysis (CFA) to formally validate the final 15-item scale. Fit indices (GFI = 0.927, CFI = 0.931, AGFI = 0.900, RMSEA = 0.058) indicated acceptable model fit, and standardized regression weights confirmed that items loaded appropriately on their intended factors. Correlations among subscales and demographic checks further supported the coherence and representativeness of the content.

In summary, the Free Will Inventory appears to show comparatively strong content validity, particularly through its inclusion of dualism alongside free will and determinism, which broadens conceptual coverage beyond earlier measures. However, difficulties in empirically separating these constructs indicate that conceptual breadth does not necessarily translate into clear psychometric distinction. Through the combination of expert review, multi-round EFA, and confirmatory factor analysis, the FWI provides formal evidence that the final scale adequately represents the intended content domain while maintaining conceptual coherence. However, empirical overlap among some subscales (e.g., dualism and free will) suggests that researchers should interpret subscale scores as reflecting related rather than fully discrete constructs.

#### 3.2.5 Construct validity

Construct validity refers to the extent to which a measure accurately captures the theoretical construct it is intended to assess and is a crucial element in evaluating any psychometric tool (Bowling and Hammond, 2008). One of the central aims in the development of the FWI was to create a measure that reflects individuals’ beliefs about free will in alignment with the major philosophical positions on the topic. Part One of the FWI is structured around three subscales - Free Will (FW), Determinism (DE), and Dualism (DU) - each designed to reflect a distinct conceptual component of free will belief.

These subscales were refined through a four-round validation process involving factor analysis. In the first round, factor analysis identified seven factors with eigenvalues above 1.0. Due to issues of overlap and ambiguity between factors, the authors reworded several items to improve clarity and theoretical distinction. In the second round, five factors emerged, but items related to dualism failed to load significantly onto any factor. The third round produced four factors, although difficulties remained in separating items cleanly across constructs, likely reflecting the challenge for lay participants in distinguishing subtle philosophical nuances. The final round of factor analysis identified eight factors with eigenvalues above 1.0; however, only three of these factors had more than one item load strongly onto them. The remaining factors were discarded due to insufficient item loading (Nadelhoffer et al., 2014).

These findings suggest that while the FWI captures three key dimensions of free will beliefs, some aspects, particularly dualism, may be more difficult to distinguish psychometrically. This could reflect either limitations in item construction or the inherently complex and overlapping nature of the constructs themselves. Additionally, the inclusion of Part Two in the FWI provides an opportunity to explore broader relationships between free will, responsibility, punishment, and metaphysical beliefs (e.g., the existence of souls or scientific determinism). However, researchers using Part Two must weigh the benefit of gaining richer conceptual insight against the risk of reduced interpretability due to conceptual overlap. Its inclusion should be guided by the specific aims of the study and the clarity of theoretical distinctions required.

A further consideration concerns not whether the FWI’s subscales are empirically separable, but what kind of belief each subscale measures. As outlined in the introduction, the FWI spans several conceptually distinct levels: metaphysical beliefs about determinism, sourcehood, and immaterial minds; psychological beliefs about choice, control, and freedom from constraint; moral beliefs about responsibility and desert; and socially relevant attitudes towards blame and punishment. These levels need not reflect a single underlying disposition, and treating them as expressions of one broad belief in free will risks conflating distinct constructs.

This concern is especially relevant to Part Two, where items concerning responsibility and punishment may reflect moral values, religiosity, political orientation, or cultural context as much as belief in free will narrowly understood. Nadelhoffer et al.’s (2014) own findings are consistent with this interpretation: several Part Two items were associated with demographic variables, including age, political ideology, and religiosity, as well as with the Part One subscales. This suggests that Part Two may be valuable for exploring the wider network of beliefs surrounding free will, but should not be interpreted as measuring free will belief alone. Indeed, Nadelhoffer et al. (2014) describe Part Two as examining relationships among beliefs about free will, choice, responsibility, punishment, souls, and scientific prediction, rather than as a scored subscale measuring a single construct.

More broadly, evidence from outside the FWI literature suggests that general belief in free will may not be the level at which free-will-related judgements are made. Monroe et al. (2017) found that manipulating people’s general free-will beliefs did not change blame and punishment judgements, whereas agent-specific ascriptions of choice capacity did. The implication is not that the FWI lacks construct validity, but that its construct validity should be understood in qualified terms. The instrument appears to measure the strength of a broad, philosophically framed set of beliefs about free will, determinism, and dualism. These scores should not be assumed to map directly onto lay concepts of free will, nor onto the moral and attitudinal judgements with which free will is often associated.

In summary, the Free Will Inventory demonstrates reasonable construct validity, although this validity should be understood in qualified terms. The repeated difficulties observed during factor-analytic refinement - particularly in isolating dualism - suggest that its constructs may be less clearly differentiated at the level of lay belief than theoretically assumed. In addition, the conceptual considerations outlined above indicate that the FWI subscales capture the strength of broad, philosophically framed beliefs, rather than providing a direct measure of lay free-will concepts or the moral and attitudinal judgements associated with them. Its subscales are therefore best interpreted as related and partially overlapping constructs, and its scores as one component of a wider network of beliefs about agency, responsibility, causation, and punishment.

#### 3.2.6 Predictive validity

Predictive validity refers to whether the test can predict future behaviour (Urbina, 2014). Regarding free will research and measurements, the question of predictive validity is challenging as there is no direct measure regarding acting in a manner accordant with having free will beliefs. However, it may be possible for data from other measures to be used to corroborate free will beliefs as determined by the FWI; it may, in principle, then be possible to ascertain whether scores on the FWI could predict future behaviour. Such measures could come in the form of observable behaviour, evidence from witnesses, and other types of reports regarding behaviours related to free will. However, obtaining such additional information in the case of free will beliefs is challenging.

Furthermore, there is no evidence to suggest that people who receive a high score on their belief in free will actually have more free will than someone who received a low score or that they act with more agency than those with lower scores (Baumeister, 2008). In fact, in some cases, even the most ardent determinists can act and live their lives as if they have free will. It is, however, believed that attitudes and beliefs towards free will could be deeply ingrained in personal identities and world views (Rychlak, 1993) and that the beliefs could have an indirect but significant impact on behavioural tendencies (Paulhus and Carey, 2011). For instance, Vohs and Schooler (2008) report that manipulating participants to doubt their free will (measured via the Free Will and Determinism scale) makes them more likely to cheat on an upcoming exam, while Shariff et al. (2014) used regression analysis to demonstrate that endorsing more mechanistic views of human behaviour (also measured via the FWD) predicts less punitive sentencing decisions. Although these findings are based on a different measure, they are relevant in illustrating the types of behavioural relationships that, in principle, could be explored using the FWI to assess its predictive validity.

Further research is needed to explore the impact of free will beliefs on behaviour in order to then investigate the predictive validity of the FWI. Nevertheless, measuring free will via the FWI can be valuable in shaping our understanding of moral reasoning, social behaviour, legal decisions, and it might even have clinical implications. Moreira-de-Oliveira et al. (2022) used the FWI to assess free will beliefs among people with obsessive-compulsive disorder and found that those with more severe symptoms appear to have a decreased belief in free will. By understanding the impact of free will beliefs, researchers and practitioners might identify and create interventions and policies that account for the role of these beliefs in both decision-making and potentially clinical settings.

In summary, the predictive validity of the FWI has not yet been empirically established, as no studies have directly examined whether FWI scores prospectively predict behaviour. While the developers of the FWI did not explicitly position the measure as a behavioural predictor, questions of predictive validity remain relevant given the frequent use of free will beliefs to interpret moral, legal, and social outcomes. Existing evidence linking free will beliefs to behaviour derives primarily from alternative measures and cannot be assumed to generalise to the FWI. Accordingly, the current utility of the FWI lies in assessing patterns of belief rather than forecasting behaviour, with predictive applications representing a potential - rather than demonstrated - avenue for future research.

## 4 Conclusion

Taken together, the evidence reviewed in this article highlights both the strengths and limitations of the Free Will Inventory as a measure of free will beliefs. Over the past 50 years, researchers have developed various tools to measure individuals’ beliefs about free will, a task complicated by the abstract, philosophical nature of the construct. The Free Will Inventory (FWI) was designed to address some of the conceptual shortcomings of earlier measures by aligning more closely with terminology and frameworks commonly used by philosophers, with the aim of enhancing construct validity.

Alongside the FAD-Plus, the FWI is now one of the most widely used instruments in free will research (Genschow et al., 2022) and has been employed across diverse cultural contexts. It measures beliefs in free will, determinism, and dualism, as well as the complex relationships between these constructs. The FWI is relatively brief, easy to administer, and has been translated into multiple languages, increasing its accessibility and potential for use in large-scale and cross-cultural studies.

While existing research supports aspects of the FWI’s reliability and validity - particularly internal consistency and concurrent validity - some areas remain underexplored. In particular, test–retest reliability and predictive validity have not yet been established. The lack of predictive research may reflect the philosophical challenge of linking free will beliefs to measurable behaviour, especially given the potential for such beliefs to fluctuate over time. In addition, the existing evidence base remains limited by the relatively narrow range of comparator measures used, the reliance on simple correlational approaches to discriminant validity, and the uneven psychometric performance of translated versions across cultural contexts.

Beyond these psychometric considerations, this review has argued that evaluating the FWI also requires attention to what the instrument measures. The FWI captures the strength of belief in free will, determinism, and dualism as these constructs are framed within philosophical debate. However, this framing may diverge from how ordinary people understand free will, which appears to centre more on unconstrained choice than on metaphysical commitments. In addition, its subscales, and particularly the responsibility- and punishment-related items in Part Two, span several conceptually distinct kinds of belief, from metaphysical claims about causation and immaterial minds to moral and attitudinal judgements about responsibility, blame, and punishment. These beliefs need not reflect a single underlying disposition. Consequently, while the FWI is conceptually grounded, its scores are best understood as indicating the strength of a broad, philosophically framed set of beliefs, rather than as a direct index of lay free-will concepts or of the moral and social judgements with which free will is often associated. Clarifying this relationship is central to the measure’s interpretability and to future evaluations of its construct validity.

Some questions also remain regarding the accessibility and interpretability of the instrument. Some items, particularly in Part Two, may be difficult for lay respondents to interpret due to the inherent complexity of the philosophical concepts being assessed. However, the developers have acknowledged these challenges and attempted to strike a balance between accessibility and conceptual rigour. This suggests that the suitability of Part Two may depend on the aims of the study and the background knowledge of the sample, with some research contexts favouring the clearer and more psychometrically established Part One subscales.

Taken together, the available evidence highlights several priorities for future research. First, studies should establish the test–retest reliability of the FWI in order to determine whether it captures relatively stable beliefs or more context-sensitive attitudes. Second, further work is needed to examine predictive validity, particularly in relation to outcomes such as moral judgement, punishment attitudes, and broader social behaviour. Third, researchers should strengthen the evidence for convergent and discriminant validity by comparing the FWI with a wider range of theoretically related measures and by applying more formal psychometric tests. Fourth, additional cross-cultural validation is needed, particularly for translated versions of the scale, to determine whether the same constructs are being captured consistently across languages and populations. Fifth, further conceptual and empirical work should clarify what level of belief the FWI captures, including the extent to which its scores reflect lay concepts of free will and whether they relate to the specific moral and punitive judgements that free-will beliefs are often assumed to underpin. Finally, future scale refinement may be warranted for philosophically dense items, especially in Part Two, to improve accessibility without sacrificing conceptual depth.

These issues also have practical implications. For researchers selecting a measure of free will beliefs, the present review suggests that the FWI is currently best suited to cross-sectional research examining individual differences in beliefs about free will, determinism, and dualism, particularly where conceptual breadth is important. At the same time, caution is warranted when using the FWI in longitudinal, intervention-based, or cross-cultural research without additional psychometric support. In applied fields such as criminal justice, education, and clinical research, where beliefs about agency, responsibility, and punishment may shape important attitudes and decisions, the FWI offers a useful framework for examining such beliefs, but its scores should not yet be treated as stable indicators or as established predictors of behaviour.

Overall, the FWI can be seen to represent a meaningful advance in the measurement of free will beliefs and demonstrates stronger theoretical and psychometric grounding than many of its predecessors. At present, it appears to be a conceptually well-grounded and moderately reliable research tool rather than a fully established instrument with comprehensive psychometric support across contexts. Further research is needed to strengthen its empirical foundation and assess its full potential as a cross-culturally robust and behaviourally relevant tool.
